# Performance of Matrix-Assisted Laser Desorption Ionization–Time of Flight Mass Spectrometry for Identifying Clinical Malassezia Isolates

**DOI:** 10.1128/JCM.01763-16

**Published:** 2016-12-28

**Authors:** Julie Denis, Marie Machouart, Florent Morio, Marcela Sabou, Catherine Kauffmann-LaCroix, Nelly Contet-Audonneau, Ermanno Candolfi, Valérie Letscher-Bru

**Affiliations:** a Laboratoire de Parasitologie et de Mycologie Médicale, Hôpitaux Universitaires de Strasbourg, Strasbourg, France; b Institut de Parasitologie et de Pathologie Tropicale, EA 7292, Fédération de Médecine Translationnelle, Université de Strasbourg, Strasbourg, France; c Structure de Parasitologie-Mycologie, Département de Microbiologie, Centre Hospitalo-Universitaire de Nancy (CHU-Nancy), Hôpitaux de Brabois, Vandœuvre-les-Nancy, France; d Laboratoire de Parasitologie-Mycologie, CHU de Nantes, Nantes, France; e EA 1155 IICiMed, UFR de Pharmacie Nantes Atlantique Universités, Nantes, France; f Laboratoire de Parasitologie-Mycologie, CHU de Poitiers, Poitiers, France; University of Manchester

**Keywords:** MALDI-TOF, Malassezia identification, ITS sequencing, ITS identification, Malassezia

## Abstract

The genus Malassezia comprises commensal yeasts on human skin. These yeasts are involved in superficial infections but are also isolated in deeper infections, such as fungemia, particularly in certain at-risk patients, such as neonates or patients with parenteral nutrition catheters. Very little is known about Malassezia epidemiology and virulence. This is due mainly to the difficulty of distinguishing species. Currently, species identification is based on morphological and biochemical characteristics. Only molecular biology techniques identify species with certainty, but they are time-consuming and expensive. The aim of this study was to develop and evaluate a matrix-assisted laser desorption ionization–time of flight (MALDI-TOF) database for identifying Malassezia species by mass spectrometry. Eighty-five Malassezia isolates from patients in three French university hospitals were investigated. Each strain was identified by internal transcribed spacer sequencing. Forty-five strains of the six species Malassezia *furfur*, M. sympodialis, M. slooffiae, M. globosa, M. restricta, and M. pachydermatis allowed the creation of a MALDI-TOF database. Forty other strains were used to test this database. All strains were identified by our Malassezia database with log scores of >2.0, according to the manufacturer's criteria. Repeatability and reproducibility tests showed a coefficient of variation of the log score values of <10%. In conclusion, our new Malassezia database allows easy, fast, and reliable identification of Malassezia species. Implementation of this database will contribute to a better, more rapid identification of Malassezia species and will be helpful in gaining a better understanding of their epidemiology.

## INTRODUCTION

The lipophilic yeasts of the genus Malassezia were first observed by Charles Robin in 1853 (1). The taxonomy of this genus has evolved since then, and it now contains 14 distinct species (2), among which 10 are commensals of the human skin: Malassezia *furfur*, M. pachydermatis, M. sympodialis, M. globosa, M. obtusa, M. slooffiae, M. restricta, M. dermatis, M. japonica, and M. yamatoensis. The four remaining species (Malassezia *nana*, M. caprae, M. equina, and M. cuniculi) are commensals of animals' skin (cats, goats, horses, and rabbits, respectively). Recently, yeasts morphologically similar to those of the genus Malassezia have been described in marine ecosystems (3). Interestingly, a study showed that Malassezia species could represent 50 to 80% of the human skin microbiota (4), with distinct geographical differences (5). For example, M. globosa is the species most frequently isolated from healthy skin in Japan and Iran (6), while in Canada the most frequently isolated species is M. sympodialis (7), and in Korea it is M. restricta (8). Some other studies have described various predominant species depending on the body site, the geographical location, and the methods used for their identification (5, 7). Malassezia species are opportunistic pathogens, causing various skin diseases, such as pityriasis versicolor, seborrheic dermatitis, or dandruff (9). Their incidence seems to be higher in some autoimmune disorders, such as psoriasis or atopic dermatitis (10–12). Although rare, some cases of fungemia and other invasive infections (peritoneal and biliary infections) (13, 14) have been described since the 1980s (13–15). These infections have no specific clinical signs and therefore remain difficult to diagnose.

Mycological diagnosis can be performed on skin or tissue samples and consists of direct examination and culture using lipid-enriched media at 32°C. Microscopic examination of clinical samples and colonies shows that small ovoid, ellipsoidal, or cylindrical blastoconidia (1.1 to 2.5 μm by 1.7 to 2.7 μm) with a large-base bud are characteristic of the genus Malassezia (2). Classical species identification comprises both biochemical and morphological studies, but this is rarely done due to the difficulty of interpreting lipophilic tests and the morphological closeness of the different species (16–18). Insufficiency of species identification contributes to the lack of epidemiological knowledge about Malassezia infections. Alternatively, molecular biology techniques, such as sequencing of large-subunit (LSU) rRNA (19) or the internal transcribed spacer (ITS) of 26S rRNA (20), can provide reliable species identification, but these techniques are usually reserved for strains from patients with deep infections.

An easy, fast, and simple method to identify Malassezia species could enhance the precise diagnosis of these infections and would provide better knowledge of the epidemiology of each species in the clinical setting. The aims of this study were (i) to develop a matrix-assisted laser desorption ionization–time of flight (MALDI-TOF) database for the identification of the main Malassezia species of clinical interest and (ii) to evaluate this database prospectively in routine practice, on clinical strains.

## RESULTS

### Database creation.

Under the conditions described in Materials and Methods, we obtained 45 main spectrum profiles (MSPs) distributed across six species: M. furfur, M. sympodialis, M. slooffiae, M. globosa, M. restricta, and M. pachydermatis.

**FIG 1:**
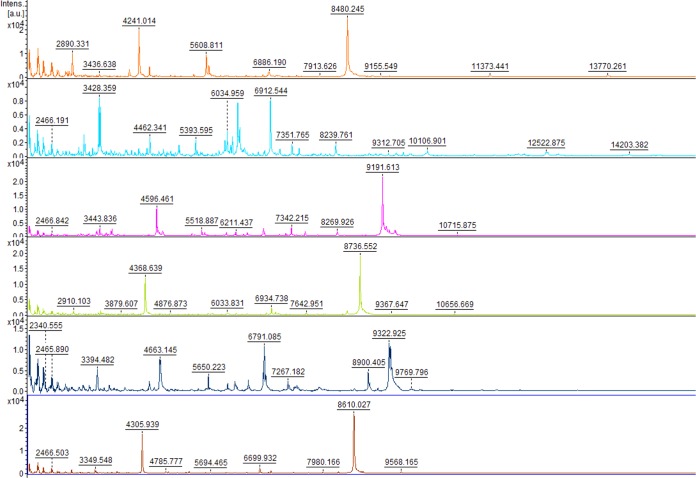
Comparison of spectra obtained for the 6 species M. slooffiae (orange), M. furfur (light blue), M. globosa (purple), M. pachydermatis (green), M. restricta (dark blue), and M. sympodialis (red).

**FIG 2:**
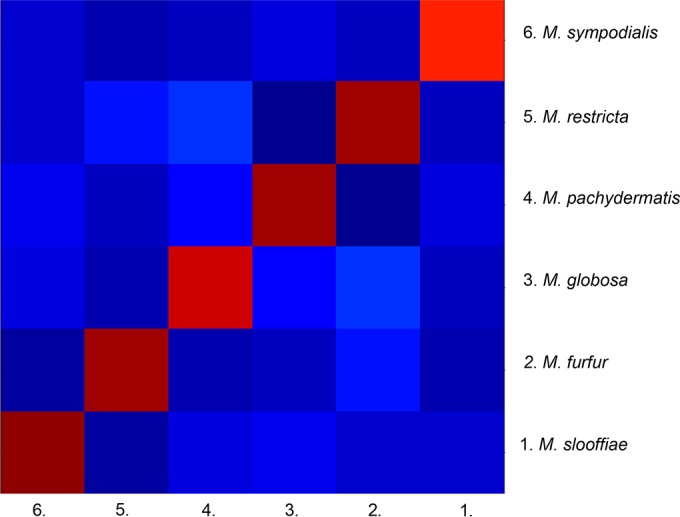
Graphical representation of CCI scores calculated for each species. Cold colors (green to blue) represent CCI scores ranging from 0 to 0.5 (weak similarity). Warm colors (red to yellow) represent CCI scores from 0.5 to 1 (high similarity).

**FIG 3:**
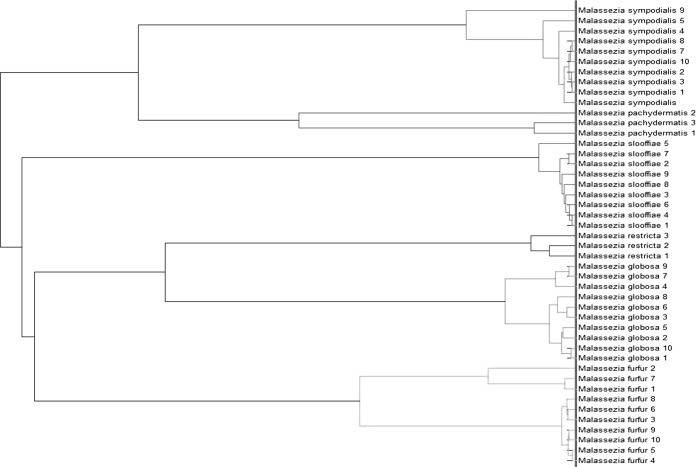
Dendrogram of the Malassezia database obtained by the MSP approach.

In the building of the database, the genus and species specificities of mass spectra were verified. To check that our spectra were genus specific, we compared the 45 MSPs obtained to Bruker Daltonics database MBT DB-5627, which contains four Malassezia strains (1 M. furfur and 3 M. pachydermatis strains). No MSPs were misidentified as non-Malassezia genera, but only one was correctly identified to the genus level: an M. pachydermatis strain with a log score of 2.13. The spectra were then compared at the species level. Comparison of the intensity and the position of the *m/z* of the spectra showed differences between the spectra of different species (Fig. 1). The composite correlation index (CCI) analysis of the spectra showed low similarity between the MSPs of different species, with a score close to zero, and high similarity within species (Fig. 2), with a score close to 1. The construction of an MSP dendrogram confirmed the distribution of the MSPs into species clusters (Fig. 3). The spectra were sufficiently discriminative between species to allow identification at the species level.

### Database test.

To test our database, we acquired the spectra of 40 additional sequenced clinical strains and compared them to both Bruker Daltonics database MBT DB-5627 and the new Malassezia database. As expected from preliminary experiments, none of the strains were identified using the Bruker MBT DB-5627 database. When compared to our Malassezia database, 100% of the strains (*n* = 40) were correctly identified at the species level, with log scores of >2.0.

For the reproducibility and repeatability of analysis and full-protocol tests, all the coefficients of variation (CVs) calculated on the log scores were lower than 10%. CVs were not significantly different according to the age of colonies within each species tested (*P*, 0.34, 0.95, and 0.77, respectively). For all species, the average CVs of the analysis and full protocol were 4% (range, 3 to 6%) and 4.9% (range, 3 to 6%), respectively. For the reproducibility tests, the average CV was 5.9% (range, 3 to 7%). When the reproducibility and repeatability CVs were compared, there were no significant differences depending on the age of the colonies (Table 1).

**TABLE 1:** Means of the coefficients of variation of the log score values of the reproducibility and repeatability tests

Age of colony (days)	Mean (range) CV (%) of log score values		
	Analysis repeatability	Full-protocol repeatability	Reproducibility
2	4.2 (3–6)	4.5 (3–6)	5.4 (2–7)
3	3.6 (3–5)	6 (5–7)	4.8 (4–8)
4	4.2 (3–6)	4.5 (4–5)	5.8 (3–9)
5	4 (3–6)	4.5 (4–5)	6.6 (5–8)

## DISCUSSION

In this study, we developed and implemented a MALDI-TOF database to identify several species of the genus Malassezia. Since Dixon medium contains Tween 40, which inhibits the MALDI-TOF signal (21–23), we decided to use subcultures on a Sabouraud medium enriched with 0.5% olive oil. These results differ from those published by Kolecka et al. (24), who succeeded in building a MALDI-TOF database for identifying Malassezia yeasts from strains cultivated on Dixon medium, possibly due to the use of another mass spectrometer. A second Malassezia database built and tested by another group (25) was obtained from cultures grown on CHROMagar Malassezia medium, which contains glycerol and a low proportion of Tween 60. Furthermore, modification of the quantities of formic acid and acetonitrile during the extraction procedure was revealed to be crucial to obtaining interpretable spectra.

Importantly, none of the 40 clinical strains tested in the present study were correctly identified with Bruker Daltonics database MBT DB-5627 even at the genus level, suggesting that this database, though licensed for clinical use, lacks performance for the genus Malassezia. This can be explained by the low number of strains included in the database (1 for M. furfur and 3 for M. pachydermatis) and by the different media used to cultivate those strains (Leeming and Notman agar modified for Malassezia species and Sabouraud medium enriched with 1% olive oil [compared to 0.5% olive oil in our medium]), since some studies have demonstrated that performance could be affected by the growth medium (26, 27).

Our Malassezia database contains 45 strains from six clinically relevant species: M. furfur, M. sympodialis, M. slooffiae, M. globosa, M. restricta, and M. pachydermatis. The MSPs obtained are genus specific, reasonably homogenous across the six species, and discriminative enough between them to allow robust Malassezia identification at the species level. All strains tested against this database were identified at the species level with a log score value of >2.0. These results are comparable with those of the other two databases published recently. The first, developed by Kolecka et al. (24), included 14 species (48 strains) and identified 84.8% of the strains tested, whereas the latter, from Yamamoto et al. (25), contained 8 species (18 strains) and identified 92.8% of the unknown strains with a log score value between 1.7 and 2.0. Although our database currently contains only six species, it remains representative of the local epidemiology of Malassezia yeasts in France. Nevertheless, further experiments are now required to implement the database with additional spectra, including those for rare species, in order to provide a better overview of Malassezia species responsible for human diseases.

The repeatability and reproducibility of the entire protocol are acceptable, with CV values of <10%, regardless of the age of the colonies within the tested range. Some studies showed that the quality of the extract is highly dependent on the quality of the culture (28). Within the genus Malassezia, the colony texture differs between species and may influence the quality of the extracts.

With 100% identification of the strains and CV values of <10%, our database can easily be used by clinical laboratories for the identification of Malassezia species. It allows for quick identification of Malassezia species, which was not possible with classical techniques such as microscopy and biochemical tests. In conclusion, we have developed a rapid and simple method for identifying Malassezia species, which promotes a better understanding of the pathogenicity of the different Malassezia species.

## MATERIALS AND METHODS

### Sample collection.

Strains isolated from skin, urinary, fecal, respiratory, and blood samples and identified as Malassezia spp. in the university hospitals of Strasbourg, Nancy, and Nantes from 2012 to 2015 were collected. First, 45 strains were used to generate the MALDI-TOF database. Forty other strains were tested and were used to evaluate the new Malassezia database (Table 2).

**TABLE 2:** Distribution and number of strains of each species included in this study

Species	No. of strains used:	
	For database generation	For clinical validation
M. furfur	10	14
M. globosa	10	4
M. restricta	3	3
M. slooffiae	9	5
M. sympodialis	10	14
M. pachydermatis	3	0
**Total**	**45**	**40**

### Strain identification.

All strains were first cultured on Dixon medium (29) at 32°C for 10 days. A microscopic morphology examination after staining with methylene blue allowed genus identification. In parallel, all strains were identified to the species level by sequencing (GATC Biotech, Germany) of the internal transcribed spacer (using primers ITS1 and ITS4) of the ribosomal DNA and comparison of the sequences obtained to those in the GenBank (http://www.ncbi.nlm.nih.gov/GenBank) and CBS (http://www.cbs.knaw.nl) databases (2, 6). The following identification criteria were used: a sequence length between 500 and 700 bp, with an E value of 0 (GenBank), a minimum overlap of 99% (CBS), and an identification concordance higher than 99%.

### MALDI-TOF MS analysis. (i) Acquisition of spectra.

Because preliminary experiments performed on a Microflex mass spectrometer (Bruker Daltonics, Germany) showed that no spectra could be acquired when the strains were cultivated on Dixon medium, we decided to subculture each strain on Sabouraud chloramphenicol agar (45.5 g Sabouraud chloramphenicol, 7.5 g Pastagar B [both from Bio-Rad, Marnes-la-Coquette, France], distilled water to 1 liter) enriched with 0.5% commercial organic olive oil. Under these conditions, mass spectra were reproducible using 2- to 5-day-old colonies and a protein extract conserved at +4°C for <48 h after extraction.

Protein was extracted as recommended by the manufacturer. Briefly, one loopful (1 μl) of yeast material was suspended in 300 μl of distilled water and 900 μl of ethanol. The suspension was mixed and was centrifuged at 13,000 × *g* for 5 min. After removal of the supernatant, the pellet was centrifuged again to eliminate the remaining ethanol. The pellet was then air dried for 10 min and was resuspended in 70% formic acid–30% acetonitrile (vol/vol). After 5 min of centrifugation at 13,000 × *g*, the pellet was removed. Then 1 μl of extracted protein, <48 h old, was transferred to a steel target, air dried, and coated with 1 μl of a matrix solution of α-cyano-4-hydroxycinnamic acid (HCCA). For spectrum acquisition, it was necessary to adapt our extraction protocol in order to standardize the quantity of yeast material used. This was difficult because of the variability of texture among the different species, particularly for M. restricta and M. slooffiae, which have drier colonies than the other species. We thus adjusted the amount of formic acid and acetonitrile to the volume of the yeast pellet obtained after washing: the volume-to-volume mixture of formic acid and acetonitrile must visually represent 1.5 times the size of the pellet (15 to 40 μl of formic acid and acetonitrile was used in this case).

Mass spectrometry analysis was performed with a Microflex mass spectrometer using Biotyper software, version 3.1. Spectra were recorded in the linear positive mode at a laser frequency of 20 Hz within a mass range from 2 to 20 kDa. The ionization source was fitted out with a delayed extraction time of 400 ns. Each spectrum was obtained from 240 shots in 40-shot steps from different positions on the target plate, and log scores were calculated. We used the manufacturer's identification criteria, which were based on the score value, as follows: <1.7, no reliable identification; between 1.7 and 2.0, genus identification; ≤2.0, species identification.

### (ii) Generation of the MALDI-TOF database for Malassezia species.

According to the manufacturer's recommendations, a minimum of 20 spectra were acquired for each strain that was used to generate the database. The spectra were uploaded on flexAnalysis, version 3.4, to smooth them and to subtract the baseline. The smoothed spectra were then analyzed by Biotyper, version 3.1. For each strain, a mass spectrum profile (MSP) was calculated and included in the database. The genus and species specificities of the spectra were analyzed using the MSP and CCI (composite correlation index) methods. The spectra obtained were also compared to Bruker Daltonics database MBT DB-5627, containing one strain of M. furfur and three strains of M. pachydermatis, to verify the genus specificity.

### (iii) MALDI-TOF database evaluation.

For each strain used for testing the database, spectra from four spots were acquired and were compared to our in-house Malassezia database and to Bruker Daltonics database MBT DB-5627. The database performance was evaluated by identification criteria, requiring correct species identification and a log score higher than 2.0. Repeatability and reproducibility were also evaluated. The repeatability of the analysis was tested for five strains—M. furfur, M. sympodialis, M. slooffiae, M. globosa, and M. restricta—on 2-, 3-, 4-, and 5-day-old colonies. For each strain, one protein extract was spotted 30 times onto the same steel target to evaluate the repeatability of the analysis. For two strains, M. furfur and M. sympodialis, the repeatability of the full protocol was tested: 15 extracts obtained at the same time, from the same culture, and from 2-, 3-, 4-, and 5-day-old colonies were deposited on the target. The complete-protocol reproducibility was also tested with three strains: M. furfur, M. sympodialis, and M. slooffiae. For each strain, 10 successive cultures were performed, and single culture extracts from 2-, 3-, 4-, and 5-day-old colonies were analyzed. The coefficient of variation (CV) of the log score values was calculated for each strain.

### Statistical analysis.

The Kruskal-Wallis test was used to compare the CVs of the repeatability and reproducibility between each pair of species. A *P* value of ≤0.05 was considered statistically significant.
